# Mechanism of Chiral-Selective Aminoacylation of an RNA Minihelix Explored by QM/MM Free-Energy Simulations

**DOI:** 10.3390/life13030722

**Published:** 2023-03-07

**Authors:** Tadashi Ando, Koji Tamura

**Affiliations:** 1 Department of Applied Electronics, Tokyo University of Science, 6-3-1 Niijuku, Katsushika-ku, Tokyo 125-8585, Japan; 2 Research Institute for Science and Technology, Tokyo University of Science, 2641 Yamazaki, Noda, Chiba 278-8510, Japan; 3 Department of Biological Science and Technology, Tokyo University of Science, 6-3-1 Niijuku, Katsushika-ku, Tokyo 125-8585, Japan

**Keywords:** homochirality of amino acids, aminoacylation, RNA, QM/MM, MD

## Abstract

Aminoacylation of a primordial RNA minihelix composed of D-ribose shows L-amino acid preference over D-amino acid without any ribozymes or enzymes. This preference in the amino acylation reaction likely plays an important role in the establishment of homochirality in L-amino acid in modern proteins. However, molecular mechanisms of the chiral selective reaction remain unsolved mainly because of difficulty in direct observation of the reaction at the molecular scale by experiments. For seeking a possible mechanism of the chiral selectivity, quantum mechanics/molecular mechanics (QM/MM) umbrella sampling molecular dynamics (MD) simulations of the aminoacylation reactions in a modeled RNA were performed to investigate differences in their free-energy profiles along the reactions for L- and D-alanine and its physicochemical origin. The reaction is initiated by approaching a 3′-oxygen of the RNA minihelix to the carbonyl carbon of an aminoacyl phosphate oligonucleotide. The QM/MM umbrella sampling MD calculations showed that the height of the free-energy barrier for L-alanine aminoacylation reaction was 17 kcal/mol, which was 9 kcal/mol lower than that for the D-alanine system. At the transition state, the distance between the negatively charged 5′-phosphate and the positively charged amino group of L-alanine was shorter than that of D-alanine, which was caused by the chirality difference of the amino acid. These results indicate that the transition state for L-alanine is more electrostatically stabilized than that for D-alanine, which would be a plausible mechanism previously unexplained for chiral selectivity in the RNA minihelix aminoacylation.

## 1. Introduction

The origin of amino acid homochirality in biological systems has long been a mystery and continues to intrigue many people. Parity violation observed in the β-decay of the nucleus suggests a slight enrichment of the L-enantiomer over the D-enantiomer (10^−11^) [1]. Studies have also suggested the enantiomeric enrichment of the L-enantiomer in an interstellar environment [2,3,4], including that caused by polarized synchrotron radiation from neutron stars [5] and enantioselective autocatalysis by chiral materials [6,7,8]. However, L-amino acids were slightly predominant in α-methyl amino acids in meteorites but not in α-H amino acids (natural components of proteins) that are more easily racemized [9]. Among these controversies, tRNA aminoacylation by aminoacyl-tRNA synthetase [10] is the most critical phenomenon because natural proteins produced in the ribosomes are composed exclusively of L-amino acid chains, which are derived from aminoacyl-tRNAs. Thus, the origin of the chiral-selective process in tRNA aminoacylation would be more important in the continuity of the evolution of biological systems on Earth. An RNA minihelix recapitulates the domain within tRNA that harbors the amino acid attachment site and may have been the progenitor of modern tRNA [11,12]. Multiple kissing-loop interaction-mediated conformational changes starting from short-hairpin RNAs possibly produced minihelix-like and then tRNA-like structures [13]. On the contrary, the duplication of minihelix-like RNA molecules could have evolved to the peptidyl transferase center on the ribosome [14,15].

**Figure 1:**
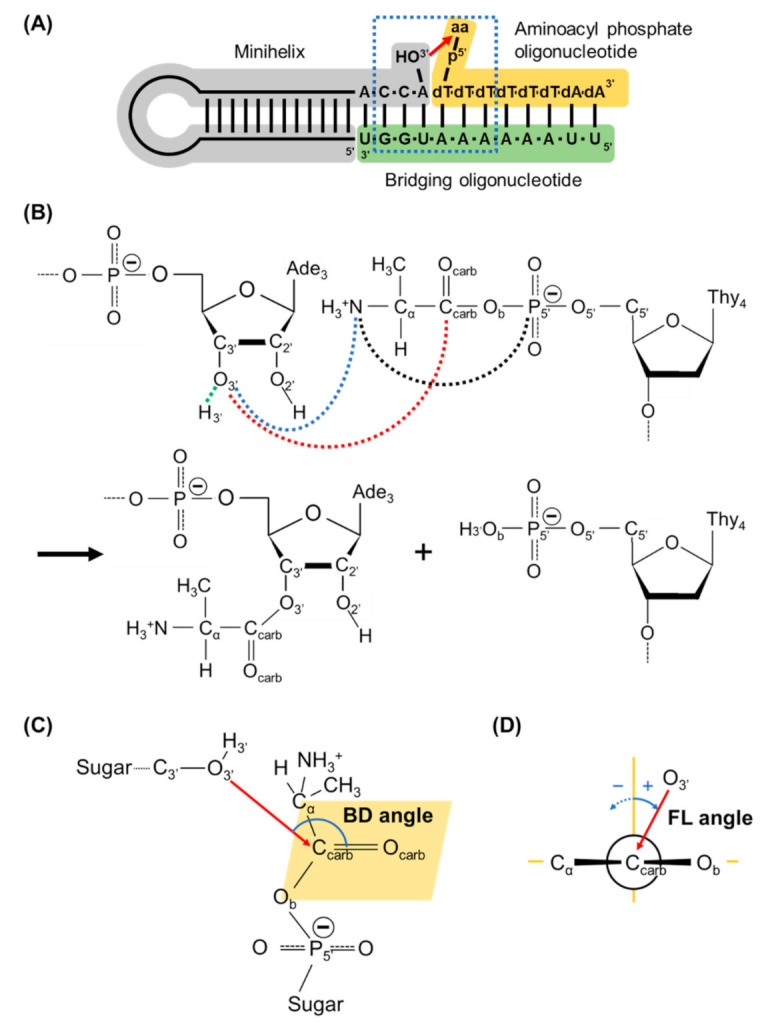
(**A**) Schematic view of aminoacylation reaction in an RNA minihelix. An aminoacyl moiety is transferred from 5′-phosphate of deoxythymine (dT) of the oligonucleotide to the 3′-hydroxyl group of adenosine (**A**) of the minihelix. Here aa represents amino acid. The 6-base pair nucleotide sequences surrounded by the blue-dotted rectangle with aa of alanine were modeled in this simulation study. (**B**) A reaction scheme for the aminoacylation found in the RNA minihelix. Distances between carbonyl carbon (C_carb_) and 3′-hydroxyl oxygen (O_3′_) atoms, *d* (C_carb_…O_3′_), and between 3′-hydrogen atom (H_3′_) and O_3′_, *d* (H_3′_ …O_3′_) are drawn with red- and green-dotted lines, respectively, which are used for defining the reaction coordinate for umbrella sampling. The distance between the nitrogen atom of alanine (N) and O_3′_, *d* (N…O_3′_), and between N and the phosphorus atom of 5′-phosphate group (P_5′_) are drawn with blue- and black-dotted lines, respectively. The abbreviations of atoms used in this study are also described. (**C**) The Bürgi–Dunitz (BD) angle is defined as the O_3′_…C_carb_=O_carb_ angle in the model. (**D**) The Flippin–Lodge (FL) angle, defined as the angle between the plane formed by O_3′_, C_carb_ and O_carb_ atoms and that perpendicular to the carbonyl plane formed by C_carb_, O_carb_, C_α_ and O_b_ atoms (shown as the orange parallelogram in (**C**)), where O_b_ is the bridging phosphate oxygen atom. In the study, the FL angle has positive values when the plane formed O_3′_, C_carb_ and O_carb_ tilts to the O_b_ atom.

Tamura and Schimmel designed a model system to achieve aminoacylation of an RNA minihelix without any help from enzymes and ribosomes, but with a bridging oligonucleotide and an aminoacyl phosphate oligonucleotide (5′-Ala-p-dT_6_dA_2_) (Figure 1A) [16], in consideration of the contemporary systems that use aminoacyl phosphate (mononucleotide) adenylates as intermediates for aminoacyl-tRNA synthesis (here, deoxynucleotides were used just for technical convenience). In this system, the L-aminoacyl-minihelix formation was preferred over that of a D-aminoacyl-minihelix formation in a ratio of approximately 4:1 [16]. RNA components with the opposite chirality (L-ribose) resulted in the preference of the formation of D-aminoacyl-minihelix over L-aminoacyl-minihelix [16]. The experimental result is important because it fits well with the “RNA world” hypothesis [17]. RNA replication could have arisen before protein synthesis and RNA could have already become chiral before the origin of protein synthesis. Thus, early tRNAs would have already used D-ribose, and this experiment shows that a D-ribose bias leads to an L-amino acid bias in the peptides that are synthesized. The chiral selectivity in the experiment was suggested to be caused by the steric clash of the amino acid side chain in the constraint of a double helical conformation [18,19]. However, in contrast to the experimental fact, the chiral-selectivity mechanism has not been fully explained.

To elucidate the possible mechanisms, so far, we have performed molecular dynamics (MD) simulations of the modeled RNA minihelix with L/D-alanine (L/D-Ala) [20]. The aminoacylation reaction was initiated by the nucleophile attack of O of 3′-OH (O_3′_) in the terminal CCA onto the trigonal unsaturated sp^2^ carbon of the carbonyl center C=O (C_carb_=O_carb_) in an acyl phosphate linkage (Figure 1B). The approaching geometry of the reaction is described by two important quantities: Bürgi–Dunitz (BD) [21,22] and Flippin–Lodge (FL) [23] angles. The BD angle was defined as the O_3′_…C_carb_=O_carb_ angle that should be approximately 105° (Figure 1C), which is measured in small-molecule crystal structures that contained amine and ketone carbonyl in the original paper of Bürgi and Dunitz [22]. The value was also validated by a theoretical study [21]. The FL angle was defined as the angle between the plane formed by O_3′_, C_carb_ and O_carb_ atoms and that perpendicular to the carbonyl plane formed by C_carb_, O_carb_, α-carbon of alanine (C_α_) and bridging phosphate oxygen (O_b_) (Figure 1D). If the size difference between C_α_ and O_b_ is small, the FL angle is simply expected to be approximately 0°. Our simulation study showed that the frequency for adopting the geometry required for the reaction with L-Ala was much higher than that with D-Ala, which was likely caused by the difference in the combination of stable dihedral angles along L- and D-alanyl phosphate backbones [20]. In general, the energy of a transition state plays a key role in determining its reaction mechanism. Therefore, for deep understanding of the chiral-selective reaction mechanism in the RNA minihelix, it is essential to describe structures and energetics along the whole reaction pathway, including the transition state of the nucleophilic substitution reaction at the trigonal carbon center. However, we could not investigate a reaction pathway in our previous study due to the use of classical MD simulation based on Newtonian mechanics [20].

Here, to elucidate the mechanism of the chiral-selective aminoacylation found in the RNA minihelix, by employing quantum mechanics/molecular mechanics (QM/MM) umbrella sampling MD simulations, we described structure and energetics along the whole pathway of the reaction. The QM/MM method provides atomically detailed structural information of reaction intermediate (s) and transition state (s) for a chemical reaction involving bond formation and breaking, which are not obtained by classical MD simulations [24]. Umbrella sampling allows us to estimate a free-energy profile along a given reaction coordinate, called a potential mean force (PMF) [25]. Hybrid QM/MM umbrella sampling MD simulations have been performed for various enzymes to investigate their reaction mechanisms [26,27,28,29,30,31]. This QM/MM umbrella sampling MD simulation study for aminoacylation reactions of the RNA minihelix provides key knowledge for explaining a chiral-selectivity mechanism observed in experiments [16].

## 2. Materials and Methods

### 2.1. Modeling of the Initial Structure for QM/MM

**Figure 2:**
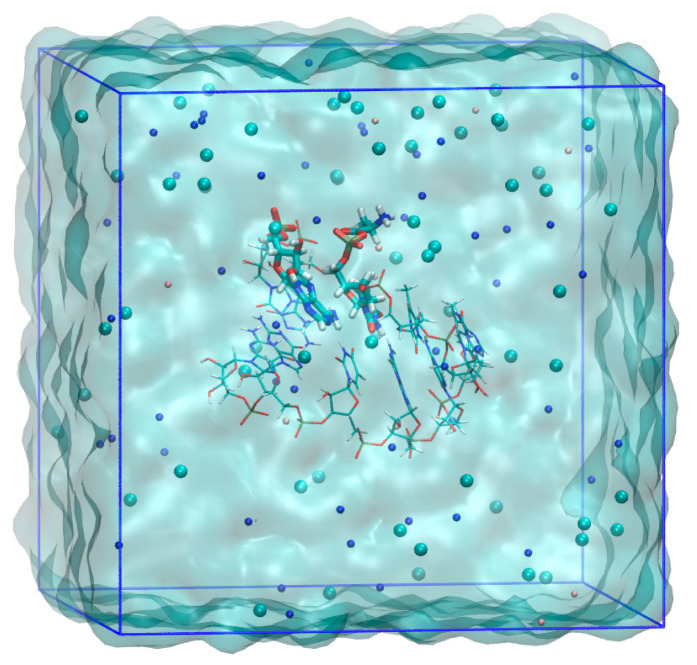
Simulation system for QM/MM umbrella sampling MD calculations for the modeled 6-base pair RNA minihelix. The image is for the D-Ala system. The residues treated with QM calculation are represented by a thick stick model. Atom colors are: oxygen (red), nitrogen (blue), carbon (cyan), phosphorus (brown), and hydrogen (white). The ions of sodium (blue), chloride (cyan) and magnesium (pink) are shown by spheres. Solvent water molecules are rendered as a cyan surface. The image was created using VMD [42].

The model of the RNA minihelix, simulation system and force field were the same as those used in our previous study [20]. Atomic structures with six base pairs centered at the reaction site composed of the RNA minihelix, a bridging oligonucleotide and 5′-Ala-p-dT_6_dA_2_ were modeled, where L/D-Ala was covalently attached at 5′-phosphate via an acyl phosphate linkage (Figure 1A). The nucleotides in the RNA minihelix consisted of D-(deoxy) ribose. The modeled RNA minihelix, which had nine excess negative charges in electron units, was solvated with approximately 2900 water molecules and 10 Mg^2+^, 63 Cl^−^ and 52 Na^+^ ions to neutralize the simulation system at approximately 1 M NaCl solution to mimics and experimental conditions (Figure 2). For DNA and RNA parts, Amber OL15 [32,33,34] and OL3 [35,36] force fields were used, respectively. For D-Ala linked to a phosphate atom, the N-terminal Ala in the Amber ff14SB force field [37] was applied, and the charge of the ester oxygen atom was assigned to −0.3079*e* to give a total charge of zero for D-Ala-dT, where *e* is the elementary charge. Amber ff14SB contained the parameters for bond, angle and dihedral involving the ester oxygen atom. The TIP3P model was used for water [38]. For monovalent and divalent ions, the parameters developed by Joung and Cheatham [39] and Li and Merz [40], respectively, were used. The system was minimized, followed by equilibration at 300 K and 1 bar conditions with the same procedures as reported in Ref. [20], except for applying distance restraints between non-hydrogen atoms involving hydrogen bonds in all base pairs to their ideal distances by a harmonic potential of the form $\frac{1}{2}k{\left(r-r_{0}\right)}^{2}$ with a force constant *k* of 1 kcal/mol/Å^2^. Subsequently, 20-ns *NPT* simulation was performed with an additional harmonic distance restraint between O_3′_ and C_carb_ atoms to 5 Å using a force constant *k* of 50 kcal/mol/Å^2^. The coordinate obtained at the end of this *NPT* simulation of the D-Ala system was used as the initial point for QM/MM MD simulations with umbrella sampling, as described below. An initial structure of the L-Ala system was generated by exchanging the coordinates between the amino and methyl groups of the D-Ala system. Therefore, the initial configurations between the L-Ala and D-Ala systems for QM/MM MD simulations only differed in their chirality. All classical MD simulations were performed using the PMEMD module in the AMBER 18 software package [41] on NVIDIA GPU RTX-2080Ti.

### 2.2. QM/MM Umbrella Sampling MD Simulations

The QM calculations were performed using Self-Consistent Charge Density Functional Tight Binding method (SCC-DFTB) [43,44,45] including the third order term of the Taylor series expansion of the DFT total energy, called DFTB3 [46], which is implemented in AMBER. The QM region comprises Ala-dT and 3′-A, whose O_3′_ attacks the carbonyl carbon C_carb_ in the acyl phosphate linkage, which is shown in Figure 2. The QM region consists of 79 atoms. All remaining atoms of the nucleic acid and solvent including ions were treated using MM with the AMBER force field.

For umbrella sampling, a reaction coordinate (RC) was chosen as the difference in the distances between H_3′_ and O_3′_, *d* (H_3′_…O_3′_) (Figure 1B, green-dotted line) and between C_carb_ and O_3′_, *d* (C_carb_…O_3′_) (Figure 1B, red-dotted line): RC = *d* (H_3′_…O_3′_) − *d* (C_carb_…O_3′_). The equilibrium distance between H_3′_ and O_3′_ atoms in the AMBER force field was 0.96 Å. Therefore, the initial coordinate for QM/MM umbrella sampling MD corresponded to the state of reaction coordinate = −4.0 Å. In total, 101 windows were computed along the reaction coordinate (an increment of reaction coordinate, ∆RC = 0.1 Å for −4.0 Å ≤ RC < −1.5 Å and 1.5 Å < RC ≤ 3.0 Å and ∆RC = 0.05 Å for −1.5 Å ≤ RC ≤ 1.5 Å) with a harmonic restraint force constant *k* of 500 kcal/mol/Å^2^. For each window, 10 ps of equilibration and 20 ps of the production run with harmonic restraints were performed, where the last coordinate of the equilibration run in the previous window was used as the starting point. A time step of 0.5 fs was used. Long-range electrostatic interactions were calculated using the particle mesh Ewald method [47,48], and short-range electrostatic and Lennard–Jones interactions were truncated at a cut-off radius of 9 Å. The temperature was controlled using the Langevin thermostat [49] at 300 K with a 1.0 ps^−1^ collision frequency. Pressure was regulated with the Berendsen algorithm [50] at 1 bar with a pressure relaxation time of 1.0 ps. Coordinates, energies and values of reaction coordinate were collected every 5 fs during the production run. Next, the weighted histogram analysis method [51,52,53] was used to compute the PMF from the simulation data for all windows with a convergence tolerance of 10^−7^. All QM/MM umbrella sampling MD simulations were performed using the SANDER module in the AMBER 18 [41].

## 3. Results

The model system by Tamura and Schimmel was composed of the extended double helix with the CCA of an RNA minihelix, an aminoacyl phosphate donor nucleotide (mimic of aminoacyl-AMP) and a bridging nucleotide [16,18]. The reaction occurs under the restriction that the nucleophile attack of O_3′_ in the terminal CCA onto C_carb_ in acyl phosphate linkage was performed with BD and FL angles to be approximately 105° and 0°, respectively. In umbrella sampling, a reaction coordinate was defined as RC = *d* (H_3′_…O_3′_) − *d* (C_carb_…O_3′_), where *d* (H_3′_…O_3′_) is the distance between H_3′_ and O_3′_ (Figure 1B, green-dotted line) and *d* (C_carb_…O_3′_) is that between C_carb_ and O_3′_ (Figure 1B, red-dotted line).

**Figure 3:**
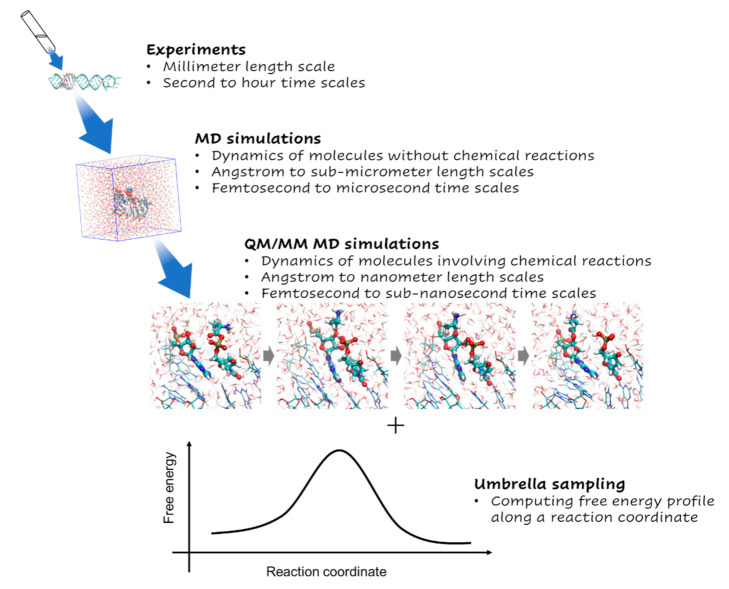
Conceptual image of QM/MM umbrella sampling MD simulations. The atom coloring scheme is the same as in Figure 2.

**Figure 4:**
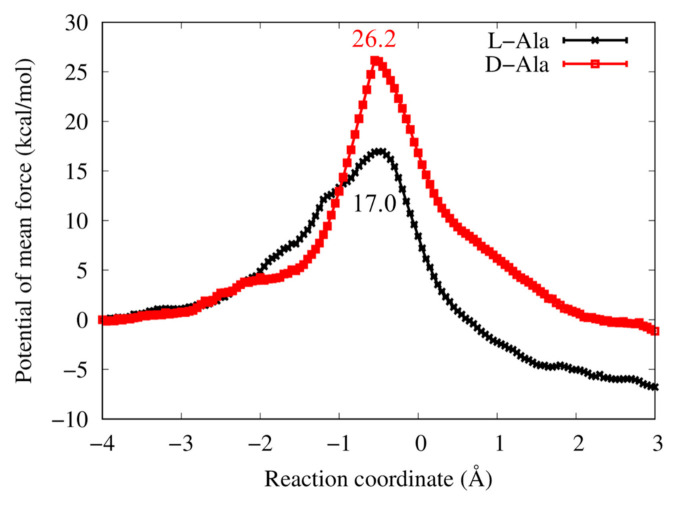
Potential of mean forces for aminoacylation reactions with L/D-Ala in the modeled RNA minihelix. The reaction coordinate (RC) is defined as follows: RC = *d* (H_3′_…O_3′_) − *d* (C_carb_…O_3′_), where *d* (H_3′_…O_3′_) is the distance between H_3′_ and O_3′_ (Figure 1B, green-dotted line) and *d* (C_carb_…O_3′_) is that between C_carb_ and O_3′_ (Figure 1B, red-dotted line). Statistical uncertainties of data point estimated by Monte Carlo bootstrap error analysis are shown with error bars, but their magnitudes are too small to see in the plot. The values in the plot are the barrier heights for L-Ala (black) and D-Ala (red) systems.

Figure 3 shows a conceptual image of the QM/MM umbrella sampling MD method used in this study. Table S1 briefly explains the simulation methods related to our method. Figure 4 shows the PMFs for aminoacylation reactions with the L/D-Ala obtained from the QM/MM umbrella sampling MD simulations. The heights of the reaction-free-energy barriers at this level of theory were 17.0 kcal/mol for L-Ala at reaction coordinate of −0.5 Å and 26.2 kcal/mol for D-Ala at reaction coordinate of −0.55 Å. The reaction barrier for L-Ala was 9 kcal/mol lower than that for D-Ala. The overall free energies were −7.0 and −1.0 kcal/mol for the L-Ala and D-Ala systems, respectively. The intermediate state was not observed for both systems.

**Figure 5:**
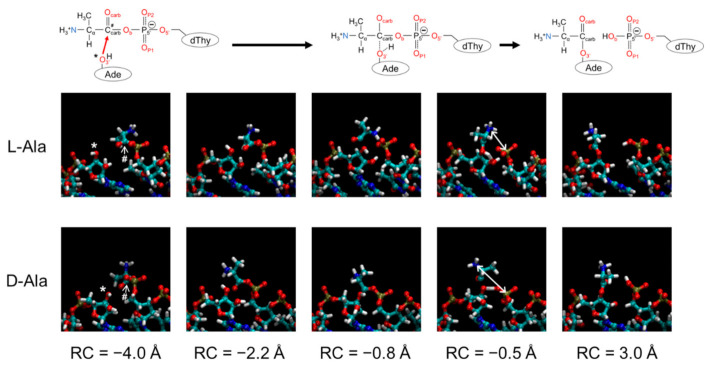
Representative structures of the aminoacylation sites for the L/D-Ala systems at various reaction coordinates (RCs). The upper panel shows chemical structures corresponding to the progress of the reaction. The 3′-oxygen (O_3′_) and carbonyl carbon (C_carb_) atoms are indicated by asterisk and hash, respectively, in snapshots at RC of −4.0 Å and the corresponding chemical structure at the upper panel. Structures at RC = −0.5 Å correspond to the transition states for L and D-Ala systems. Distances between nitrogen atom of amino group in alanine and phosphorous atom of 5′-phosphate group are shown with white arrows in the snapshots at the transition states. The coloring scheme in the snapshots is the same as in Figure 3. In the chemical structures at the upper panel, oxygen and nitrogen atoms are drawn in red and blue, respectively, and carbon, phosphorus, and hydrogen atoms are drawn in black.

Representative geometries of the aminoacylation sites for the L/D-Ala systems at various reaction coordinates are shown in Figure 5. Movies of the simulations throughout the whole reaction coordinates are also provided in Supplementary Materials (Movie S1 and Movie S2 for the L-Ala and D-Ala systems, respectively). The L/D-Ala system started with similar coordinates (reaction coordinate of −4.0 Å), and no distinct difference in coordinates around the reaction site was observed between them, except for the chirality of the terminal Ala. The overall structures of the L-/D-Ala system after the complete transfer of alanine moieties from 5′-dT to 3′-A (reaction coordinate of 3.0 Å) were also comparable. For both systems, the H_3′_ atom transferred from 3′-oxygen to one of the oxygens in the 5′-end phosphate group simultaneously with the nucleophile attack of O_3′_.

**Figure 6:**
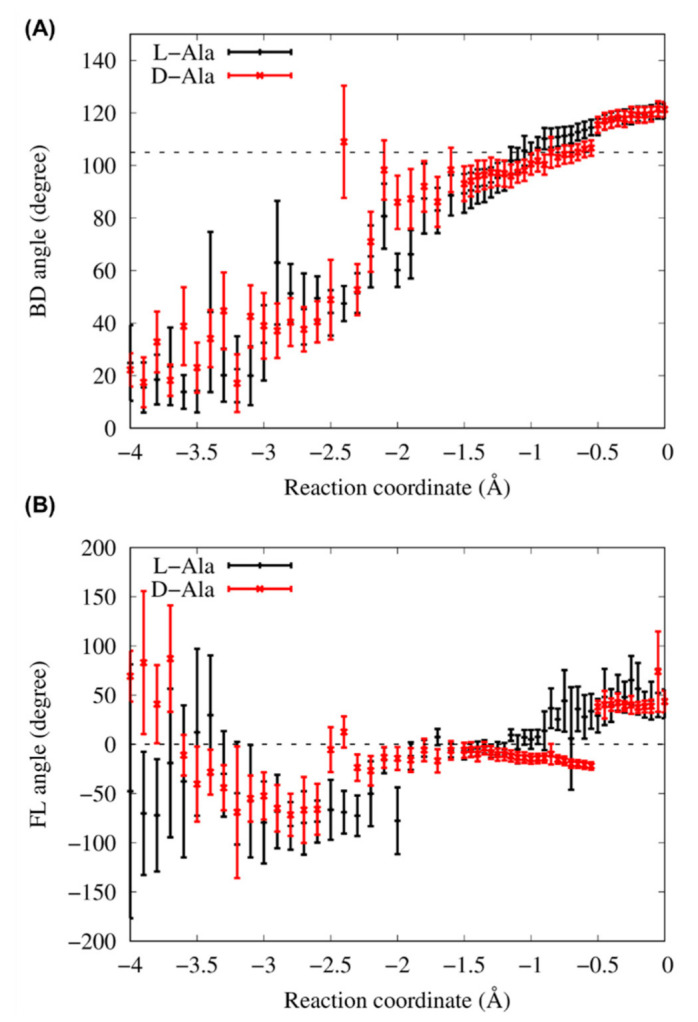
(**A**) Bürgi–Dunitz (BD) angles averaged in each window as a function of the reaction coordinate from −4 to 0 Å. The black broken line corresponds to the angle of 105°, which is measured in small-molecule crystal structures that contained amine and ketone carbonyl in the original paper of Bürgi and Dunitz [21]. (**B**) Flippin–Lodge (FL) angles averaged in each window as a function of the reaction coordinate from −4 to 0 Å. An angle of 0° is indicated by the black broken line. Error bars in (**A**) and (**B**) indicate the standard deviations.

Figure 6A,B shows the BD and FL angles, respectively, averaged over each umbrella window for reaction coordinate of −4 to 0 Å. In this analysis, the FL angle has positive values when the plane formed O_3′_, C_carb_ and O_carb_ tilts to the bridging phosphate oxygen O_b_. The BD angles gradually increased to the experimentally measured value of 105° just before the transition states, with a reaction coordinate of −1.0 Å for the L- and D-Ala systems. For the FL angles in both systems, the values fluctuated approximately 0° with small deviations for reaction coordinate of −2 to −1 Å. The simulation models used in this study well reproduced the restrictions of BD and FL angles for the aminoacylation reaction.

**Figure 7:**
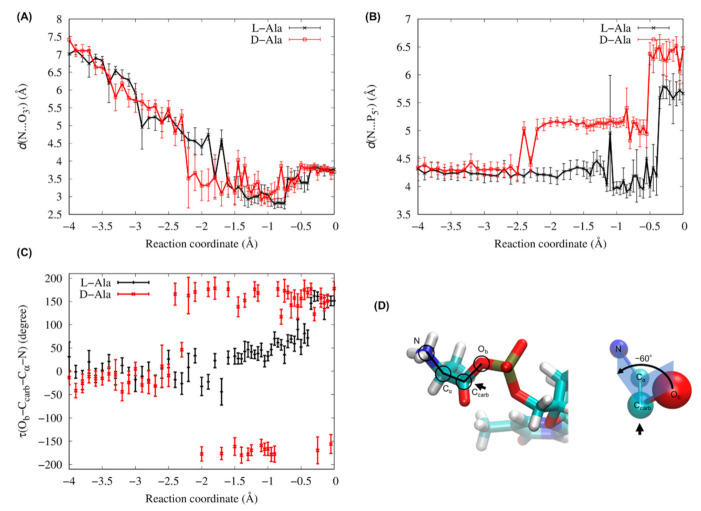
(**A**) Distances between N and O_3′_ atoms (*d* (N…O_3′_) shown in Figure 1B with blue-dotted line), (**B**) distance between N and P_5′_ atoms (*d* (N…P_5′_) shown in Figure 1B with black-dotted line) (**C**) dihedral angle for O_b_-C_carb_-C_α_-N (*τ* (O_b_-C_carb_-C_α_-N)) averaged in each window as a function of the reaction coordinate (RC) from −4 to 0 Å. (**D**) Schematic representation of the dihedral angle *τ* (O_b_-C_carb_-C_α_-N), defining the rotation of bond O_b_-C_carb_ around C_carb_-C_α_ with respect to C_α_-N. A positive sign corresponds to a clockwise rotation. In this figure, the *τ* (O_b_-C_carb_-C_α_-N) is −60°. The coloring scheme is the same as in Figure 3. Error bars in (**A**), (**B**) and (**C**) indicate the standard deviations.

To quantify structural differences between L-/D-Ala systems, three geometrical features around the active site were measured. The first measure is the distance between the nitrogen atom N of the positively charged amino group and the O_3′_ atom of the relatively high electronegative hydroxyl group, *d* (N…O_3′_) (Figure 1B, blue-dotted line). The second measure is the distance between the N atom and the P_5′_ atom of the negatively charged 5′-phospate group, *d* (N…P_5′_) (Figure 1B, black-dotted line). The third measure is a dihedral angle defined by O_b_-C_carb_-C_α_-N, *τ* (O_b_-C_carb_-C_α_-N). Figure 7 shows changes in *d* (N…O_3′_), *d* (N…P_5′_) and *τ* (O_b_-C_carb_-C_α_-N) along the reaction coordinate. These values are averaged over each umbrella window for a reaction coordinate of −4 to 0 Å. Until reaction coordinate of −2.5 Å, the trajectories and absolute values of the measures for the L-/D-Ala systems were comparable. At reaction coordinate of −2.2 Å (*d* (C_carb_…O_3′_) ≈ 3.2 Å), the averaged value of *d* (N…O_3′_) for the D-Ala system suddenly dropped from 5 Å to 3.5 Å (Figure 7A), which was coupled with a rotation of the dihedral angle *τ* (O_b_-C_carb_-C_α_-N) from 0° (*cis* conformation) to ±180° (*trans* conformation) (Figure 7C). Contrarily, for the L-Ala system, the distance *d* (N…O_3′_) gradually decreased until reaction coordinate of −0.75 Å (Figure 7A), and the dihedral angle *τ* (O_b_-C_carb_-C_α_-N) also gradually rotated from 0 to 60° during the reaction coordinate from −2.2 to −0.5 Å (Figure 7C). As a result of the difference in the mode of conformational changes, the *d* (N…O_3′_) of the D-Ala system was ~1 Å shorter than that of the L-Ala system during reaction coordinate of −2.3 to −1.6 Å; however, after reaction coordinate of −1.6 Å, the *d* (N…O_3′_) for the L-Ala system was slightly but clearly shorter than that for the D-Ala system, which continued until the transition states at reaction coordinate ≈ −0.5 Å. For the *d* (N…P_5′_), its difference between L-Ala and D-Ala systems is more evident (Figure 7B). Until reaction coordinate of −2.5 Å, the *d* (N…P_5′_) of the L-/D-Ala systems were 4.2–4.3 Å. For the D-Ala system, the averaged value of *d* (N…P_5′_) increased to 5.1 Å at reaction coordinate of −2.2 Å, which was also coupled with the *cis*/*trans* isomerization of the dihedral angle *τ* (O_b_-C_carb_-C_α_-N) (Figure 7C). The distance for D-Ala system remained around 5.1 Å until the transition state at reaction coordinate of −0.55 Å (See also Figure 5 showing the *d* (N…P_5′_) at the transition state). After passing the transition state, the *d* (N…P_5′_) in D-Ala system jumped up to 6.3 Å. On the other hand, the *d* (N…P_5′_) in L-Ala system stayed around 4.25 Å until reaction coordinate of −1.1 Å (Figure 7B). Around the transition state, the *d* (N…P_5′_) in L-Ala system, which is shown in Figure 5, fluctuated around 4.0 Å. Therefore, the distance between positively charged amino group and the negatively charged 5′-phosphate group of L-Ala system is approximately 1.1 Å shorter than that of D-Ala system at their transition states.

## 4. Discussion

In the original experiments by Tamura and Schimmel, the chiral preference for L-amino acid in the RNA minihelix was observed not only for Ala but also for Leu and Phe with approximately fourfold selectivity, which was measured by the ratio of the product formed at 30 min of reaction time [16]. As discussed in Ref. [18], an energetic difference of <1 kcal/mol in the rate-determining step is sufficient to give the fourfold preference for L- vs. D-specific aminoacylation. The calculated energy difference of 9 kcal/mol at the transition state (reaction coordinate ≈ −0.5 Å) was too large compared with the experimental estimation (Figure 4); however, the chiral preference estimated by the QM/MM simulation study is qualitatively consistent with the experimental results. The primary goal of our simulations at the SCC-DFTB3 level of theory was to provide qualitative results to investigate chiral-selectivity mechanisms, and we cannot expect that the absolute quantitative results are comparable to experimental results. Therefore, our simulation results would reflect the experimental facts.

The BD and FL angles define the approaching geometry of the nucleophilic oxygen O_3′_ atom toward the electrophile carbonyl carbon C_carb_ atom in the aminoacylation reaction, which are described by the relative positions of O_3′_, C_carb_, O_carb_, Cα and Ob atoms. In QM/MM MD simulations, both angles were gradually adopting their adequate values for the reaction until the transition states L- and D-Ala systems (Figure 6). Therefore, the observed free-energy difference between the L- and D-Ala systems at the transition state is not attributed to the local geometry difference described by only these atom positions but to the effects of surrounding atoms and functional groups around the reactive carbonyl group.

We measured the distances between the N atom of the positively charged amino group in Ala and the O_3′_ atom of the relatively high electronegative hydroxyl group, *d* (N…O_3′_), and between the N atom and P_5′_ atom of negatively charged 5′-phosphate group, *d* (N…P_5′_), to find the structural origin that generates the energy difference at the transition state between L- and D-Ala systems. Electrostatic interactions between the amino group and the negatively charged groups could largely contribute to the transition state energy. At the transition states for L-Ala (reaction coordinate of −0.5 Å) and D-Ala (reaction coordinate of −0.55 Å), the *d* (N…O_3′_) and *d* (N…P_5′_) for the L-Ala system was roughly 0.5 and 1.1 Å, respectively, shorter than those for the D-Ala system (Figure 5 and Figure 7A,B). Therefore, the transition state would be more stabilized in the L-Ala system than in the D-Ala system. This is a possible mechanism for the chiral selectivity in the RNA minihelix found in the QM/MM umbrella sampling MD simulations.

The homochirality of proteins and nucleic acids is intrinsically required in their structures: the secondary structures of proteins, α-helix and β-sheet, are likely to be formed only if the constituent amino acids are homochiral (all-L or all-D) [54], and a template-directed elongation of nucleotide chains occurs properly only when the ribose sugars of the template are homochiral [55,56]. However, why natural proteins and nucleic acids are composed of L-amino acids and D-sugars, respectively, remains unclear. It has been shown that oligomerization of all possible combinations of short homochiral and heterochiral RNA diastereomers proceeded chiral-selectively [57], forming two libraries consisting of equal amounts of homochiral all-L and all-D RNAs. However, symmetry breaking occurs inevitably because the number of possible sequences with growing oligomer length is beyond the number of actually formed sequences. D-ribose RNA would have been selected by chance due to an important chemical ability acquired through the symmetry breaking [19]. Furthermore, although the hypothesis that amino acids were chirally biased first and that the chiral bias in RNA arose secondarily cannot also be denied, ribose has four asymmetric centers (C_1′_, C_2′_, C_3′_ and C_4′_), and Watson-Crick helices can be formed properly if any two of the three asymmetric carbons (C_1′_, C_3′_ and C_4′_) are correctly positioned relative to each other [58]. In considering the complex set of asymmetric centers in ribose, the probability that L-amino acids could have selected proper formation of the chain of D-ribose with proper configurations linked through 5′-3′ phosphodiester bond would be quite low [59]. The discovery of ribozymes raised the possibility of the “RNA world” as a plausible stage in Earth’s history [17]. Thus, the transition from the putative “RNA world” to the “protein world” would have been a key step [14].

In the Tamura and Schimmel model, the RNA chirality has been shown to correlate with recognition of chiral amino acids through RNA aminoacylation: D-ribose RNA aminoacylates L-amino acids, whereas L-ribose RNA aminoacylates D-amino acids [16]. All tRNA molecules possess a single-stranded CCA sequence at their 3′-ends [14]. Although it has been proposed that tRNA-like structural motifs with CCA first evolved as 3′-terminal tags in RNA genomes for replication in the “RNA world” [60], the CCA would have also been a target for tRNA charging by the interactions with other oligomers. The primordial tRNAs could have been somewhat charged by transferring the amino acid from another oligomer as shown in this model. In this stage, a D-ribose based “RNA world” would have already been formed. In fact, an amino acid activation ribozyme was selected in vitro [61] and this reaction could have happened evolutionarily. However, in terms of the continuity of the biological evolution toward peptide synthesis on the ribosome, the transfer of amino acid from phosphate (in the form of acyl phosphate) to hydroxyl group (in the form of ester) would have been necessary. Although the chiral-selective experiments using mutants [18] and MD simulations [20] suggested the steric clash of the side chain of amino acids in the original chiral-selective model reaction, the quantum mechanical calculations must be included to provide a clear energetics of the proposed mechanism.

Thus, the present attempt was conducted, and finally, the energetic and structural differences of the active sites of the L-/D-Ala systems at the transition states were observed in our simulations. An origin that causes differences would be the chirality of amino acids. The differences in *d* (N…O_3′_) and *d* (N…P_5′_) at the transition states were initially generated by the *cis* to *trans* transition of O_b_-C_carb_-C_α_-N dihedral angle at a reaction coordinate of −2.2 Å only in the D-Ala system (Figure 7). Chirality may perturb the rotational probability of the dihedral angle.

## 5. Conclusions

Chiral-selective aminoacylation in the primordial RNA minihelix is a key reaction for homochirality of L-amino acid. In this study, we used QM/MM MD simulation techniques and explored atomistic mechanisms of the chiral-selective aminoacylation observed in experiments. Our computational study presented the model mechanism that the L-Ala moiety stabilizes the transition state more than D-Ala, resulting in L-Ala preference in the aminoacylation reaction in the RNA. For a more detailed mechanism, longer and larger QM/MM simulation studies with high-level QM theory are necessary. Specifically, the inclusion of solvent atoms around the reaction site as the QM region would be important for more quantitative evaluations of the height of the barrier at transition states, which is our further study. The computational studies presented here highlight that the QM/MM MD simulations have the potential to solve evolutionary problems in terms of molecular quantum mechanics.

## Data Availability

The data presented in this study are available from the corresponding authors upon reasonable request.

## Supplementary Materials

The following supporting information can be downloaded at: https://www.mdpi.com/article/10.3390/life13030722/s1. Table S1: Brief explanations for the methods related with the hybrid QM/MM umbrella sampling MD. Movie S1: QM/MM umbrella sampling MD simulation for the L-Ala system. Movie S2: QM/MM umbrella sampling MD simulation for the D-Ala system.
