# Supplementary material for: Mechanism of Chiral-Selective Aminoacylation of an RNA Minihelix Explored by QM/MM Free-Energy Simulations
Source: Life (Basel). 2023 Mar 7;13(3):722. doi: 10.3390/life13030722 (PMC10057131; doi:10.3390/life13030722)
Supplement: Supplementary file 1 [file life-13-00722-s001.zip › SI.pdf]

Supplementary Information

Table S1. Brief explanations for the methods related with the hybrid QM/MM umbrella sampling MD

**Molecular mechanics (MM)**<br>The potential energy with respect to the atom nuclei of the molecular system is approximated by empirical energy functions with force field parameters, where the electrons are implicitly regarded.
**Quantum mechanics (QM)**<br>The motion of the atom nuclei and electrons of the molecular system is described by the Schrödinger wave equation.
**Molecular dynamics (MD)**<br>A molecular simulation method that generates successive configurations of the system by integrating Newton's equations of motion, where the forces on the particles/atoms are calculated by MM or QM.
**Classical MD**<br>MD simulation with empirical MM potential. Simulating long-time dynamics of molecular systems is generally possible. Chemical reactions involving bond formation and breaking cannot be handled.
**QM MD**<br>MD simulation with forces calculated by QM. If QM calculations are performed from first principles, the method is called *ab initio* MD. Chemical reactions involving bond formation and breaking can be described. Simulation systems are limited to a small size because of its high computational costs.
**QM/MM MD**<br>MD simulation that treats a particular region of interest with QM and the surrounding environment with MM potentials.
**Umbrella sampling**<br>One of the methods that calculate free energy profile along a predefined reaction coordinate (RC), also called potential of mean force (PFM). The method attempts to efficiently sample energetically separated regions in phase space by adding a biasing potential to the original potential function. To ensure sampling in all regions along the RC, a harmonic biasing potential is usually applied in a series of windows along the RC. The PFM can be evaluated from the whole series of umbrella sampling data using the weighted histogram analysis method (WHAM).

Movie S1. QM/MM umbrella sampling MD for the L-Ala system

Movie S2. QM/MM umbrella sampling MD for the D-Ala system
